# Divergent modulation of Rho‐kinase and Ca^2+^ influx pathways by Src family kinases and focal adhesion kinase in airway smooth muscle

**DOI:** 10.1111/bph.13313

**Published:** 2015-10-23

**Authors:** Yasin Shaifta, Nneka Irechukwu, Jesus Prieto‐Lloret, Charles E MacKay, Keisha A Marchon, Jeremy P T Ward, Greg A Knock

**Affiliations:** ^1^ Division of Asthma, Allergy and Lung Biology, Faculty of Life Sciences and MedicineKing's College LondonLondonUK

## Abstract

**Background and Purpose:**

The importance of tyrosine kinases in airway smooth muscle (ASM) contraction is not fully understood. The aim of this study was to investigate the role of Src‐family kinases (SrcFK) and focal adhesion kinase (FAK) in GPCR‐mediated ASM contraction and associated signalling events.

**Experimental Approach:**

Contraction was recorded in intact or α‐toxin permeabilized rat bronchioles. Phosphorylation of SrcFK, FAK, myosin light‐chain‐20 (MLC_20_) and myosin phosphatase targeting subunit‐1 (MYPT‐1) was evaluated in cultured human ASM cells (hASMC). [Ca^2+^]_i_ was evaluated in Fura‐2 loaded hASMC. Responses to carbachol (CCh) and bradykinin (BK) and the contribution of SrcFK and FAK to these responses were determined.

**Key Results:**

Contractile responses in intact bronchioles were inhibited by antagonists of SrcFK, FAK and Rho‐kinase, while after α‐toxin permeabilization, they were sensitive to inhibition of SrcFK and Rho‐kinase, but not FAK. CCh and BK increased phosphorylation of MYPT‐1 and MLC_20_ and auto‐phosphorylation of SrcFK and FAK. MYPT‐1 phosphorylation was sensitive to inhibition of Rho‐kinase and SrcFK, but not FAK. Contraction induced by SR Ca^2+^ depletion and equivalent [Ca^2+^]_i_ responses in hASMC were sensitive to inhibition of both SrcFK and FAK, while depolarization‐induced contraction was sensitive to FAK inhibition only. SrcFK auto‐phosphorylation was partially FAK‐dependent, while FAK auto‐phosphorylation was SrcFK‐independent.

**Conclusions and Implications:**

SrcFK mediates Ca^2+^‐sensitization in ASM, while SrcFK and FAK together and individually influence multiple Ca^2+^ influx pathways. Tyrosine phosphorylation is therefore a key upstream signalling event in ASM contraction and may be a viable target for modulating ASM tone in respiratory disease.

**Abbreviations**
ASM: airway smooth muscle
hASMC: cultured human airway smooth muscle cells
KPSS: PSS with 80 mM equimolar substitution of Na^+^ for K^+^
MLC_20_: myosin light‐chain 20 KDa subunit
MLCP: myosin light‐chain phosphatase
MYPT‐1: myosin phosphatase targeting subunit‐1
ROCE: receptor‐operated Ca^2+^ entry
SOCE: store‐operated Ca^2+^ entry
VOCE: voltage‐operated Ca^2+^ entry

## Tables of Links

**Table:** 

**TARGETS**	
**Ion channels** *^a^*	**Enzymes** *^b^*
Store‐operated Ca2+ channels	FAK
Voltage‐gated Ca2+ channels	MLCK
	PYK2
	Src family kinases
	Rho‐kinase (ROCK)

**LIGANDS**	
Bradykinin (BK)	GTP
Carbachol (CCh)	Nifedipine
Cyclopiazonic acid (CPA)	Y27632

## Introduction

Airway smooth muscle (ASM) tone is subject to regulation by cholinergic, catecholamine and NANC neurotransmitters as well as local inflammatory mediators. In healthy airways, muscle tone is normally low, providing a low resistance path for airflow. However, contraction may be enhanced in response to chemical irritants or allergens, particularly in lower respiratory tract bronchioles (Gilbert and Auchincloss, 1989; Pinelli *et al.,* 2009). In asthma, airway resistance is increased, partly due to increased basal tone and hypersensitivity to constrictor stimuli in these bronchioles (Doeing and Solway, 2013; Meurs *et al.,* 2008).

Smooth muscle contractile force depends on the degree of myosin light‐chain‐20 (MLC_20_) phosphorylation, which is in turn determined by the balance between Ca^2+^‐dependent activation of myosin light‐chain kinase (MLCK) and Ca^2+^‐independent inhibition of myosin light‐chain phosphatase (MLCP), as well as the formation and recruitment of myofilaments (Gunst *et al.,* 2003; Somlyo and Somlyo, 2003). Increases in [Ca^2+^]_i_ result from Ca^2+^ release from the sarco‐endoplasmic reticulum (SR) and a combination of Ca^2+^ entry through receptor‐operated, store‐operated and voltage‐operated Ca^2+^ channels (ROCE, SOCE and VOCE, respectively). Inhibition of MLCP occurs via phosphorylation of myosin phosphatase targeting subunit‐1 (MYPT‐1), primarily by Rho‐kinase (Feng *et al.,* 1999), resulting in a further increase in MLC_20_ phosphorylation and contraction without the need for a further increase in [Ca^2+^]_i_ (Somlyo and Somlyo, 2003). Although it is likely that bronchoconstrictors act via a combination of the above pathways, the precise mechanisms through which Ca^2+^ influx and Rho‐kinase activity are mediated by GPCRs are not fully understood.

Src family kinases (SrcFK) and focal adhesion kinase (FAK) are widely expressed non‐receptor tyrosine kinases (TKs) important in many aspects of cellular function, being activated in response to various stimuli including growth factors, GPCRs, reactive oxygen species and adhesion. SrcFK and FAK are often described as being mutually dependent or reciprocally activated, especially when associated with integrin engagement and/or growth factor receptor activation (Owen *et al.,* 1999; Ishigaki *et al.,* 2011). An effect of TKs on ASM tone was first suggested by the relaxant effect of non‐selective tyrosine kinase inhibitors on rat isolated bronchioles (Chopra *et al.,* 1997). Subsequently, selective inhibition of SrcFK and FAK was shown to depress GPCR‐induced contraction in human, rodent or canine upper airways (Tang and Gunst, 2001; Katsumoto *et al.,* 2013). FAK was linked to elevated [Ca^2+^]_i_ in response to various stimuli in trachea, but the relative influence of the kinase on VOCE, ROCE or SOCE or on Rho‐kinase was not determined (Tang *et al.,* 1999; Tang and Gunst, 2001). SrcFKs have been identified as upstream mediators of Rho‐kinase in vascular smooth muscle (Nakao *et al.,* 2002; Knock *et al.,* 2008), but neither this relationship nor the influence of SrcFK on GPCR [Ca^2+^]_i_ responses has yet been examined in ASM. To our knowledge, only one previous study has examined the involvement of SrcFK or FAK specifically in the contraction of intralobar bronchioles, and this was limited to the role of SrcFK in mediating sensitization of rat bronchioles to muscarinic agonists (Sakai *et al.,* 2010).

In this study, we hypothesized that SrcFK and FAK mediate GPCR‐induced ASM contraction via multiple signalling pathways and examined their influence on Rho‐kinase‐dependent MLCP inhibition/Ca^2+^‐sensitization and on SOCE/ROCE and VOCE Ca^2+^ entry pathways, in intra‐lobar bronchioles of rat and cultured human ASM cells (hASMC). We found that SrcFK, most likely c‐Src itself, modulate Rho‐kinase dependent Ca^2+^‐sensitization, but FAK does not, and that the two tyrosine kinases differentially regulate SOCE/ROCE and VOCE. We also suggest the existence of two subpopulations of GPCR‐activated SrcFK, one being FAK‐dependent and the other FAK‐independent.

## Methods

### Rats and tension measurement by wire myography

All animal care and experimental procedures complied with UK legislation under the Animals (Scientific Procedures) Act 1986 Amendment Regulations (SI 2012/3039) and were deemed to be as humane as possible. All results involving animals are reported in accordance with the ARRIVE guidelines for reporting experiments involving animals (McGrath *et al.,* 2010). A total of 98 rats were used. Male Wistar rats (~250 g) had free access to food and water and were maintained on a 12:12 h light/dark schedule. The rats were killed by an i.p. injection of sodium pentobarbital and the lungs and trachea were immediately removed. First or second‐order intralobar bronchioles (~2 mm length) were dissected free of surrounding parenchyma and mounted on a wire myograph (DMT.dk), bathed in PSS (in mM: 118 NaCl; 24 NaHCO_3_; 1 MgSO_4_; 4 KCl; 5.56 glucose; 0.435 NaH_2_PO_4_; 1.8 CaCl_2_, pH 7.4), gassed with 95% air, 5% CO_2_ at 37°C. Bronchioles were incrementally stretched and alternately exposed to PSS containing 80 mM [K^+^] (equimolar substitution for Na^+^, KPSS) until the point on the length tension curve at which muscle length was optimum for active tension development was achieved, as described previously (Moir *et al.,* 2003). Viability for contraction experiments was confirmed by a response of at least 3 mN to the last challenge with KPSS. Bronchiole internal diameter after stretch was typically in the range 300–800 µm.

Specific examination of the Rho‐kinase dependent Ca^2+^‐sensitization component of contraction was achieved by permeabilizing myograph‐mounted bronchioles with α‐haemolysin (α‐toxin). PSS was first exchanged for relaxing solution (pCa = 10, in mM: 200 PIPES; 100 Mg(M_s_)_2_; 1000 KM_S_; 100 K_2_EGTA; 5 Na_2_ATP; 10 Na_2_creatine phosphate, pH 7.1), gassed with air at 26°C. α‐toxin (60 µg ml^‐1^) was then applied in relaxing solution with pCa raised to 6.7 for 30 min, permeabilization being confirmed by the development of active tension. pCa was adjusted via proportionate substitution of K_2_EGTA for CaEGTA, with 100 CaEGTA, 0 K_2_EGTA being equivalent to pCa4.5. Contractile responses to bronchoconstrictors were conducted at pCa 6.5 (~300 nM [Ca^2+^]), which induced a contraction equivalent to 10‐20% of that achieved by pCa 4.5. GTP 1 μM and 10 μM cyclopiazonic acid (CPA) were included to support G‐protein signalling and to prevent the influence of SR Ca^2+^ release on contraction respectively.

### Human tissue and cell culture

Donations of human tissue were obtained following written informed consent and with the approval of the South East London Research Ethics Committee, REC reference number 10/H0804/66. All clinical procedures conformed to the standards set by the latest Declaration of Helsinki. hASMC were obtained from healthy volunteers (*n* = 11; 7 women, 4 males; age range 22–53 years; life‐long absence of respiratory symptoms; lung functions within normal limits) by deep endobronchial biopsy. ASM bundles were bathed in DMEM containing 10% FBS, l‐glutamine (2 mM), sodium pyruvate (1 mM), non‐essential amino acids and amphotericin B (2 µg ml^‐1^), and subjected to enzymatic digestion in nominally Ca^2+^‐free HEPES buffer containing: 5.56 mM glucose, 2 mg ml^‐1^ collagenase Type XI, 1 mg ml^‐1^ papaine, 1 mg ml^‐1^ trypsin inhibitor and 1 mM DTT, for 30 min at 37°C. Cells were then dispersed into culture flasks containing DMEM (plus supplements) and incubated at 37°C, pH 7.4_._ Smooth muscle phenotype was confirmed by positive staining with anti‐smooth muscle α‐actin, anti‐desmin and anti‐calponin, with Alexa Fluor®488 labelled secondary antibody (Lifetechnologies) and with TRITC‐labelled phalloidin to confirm the presence of stress fibres in resting cells (Supporting Information Fig. S1). Cells were used for experiments at passages 4–9, grown to confluence and serum starved for 7 days in DMEM plus supplements, and the addition of 1% BSA, 5 µg ml^‐1^ transferrin, 1 μM insulin and 100 μM ascorbate.

### siRNA design and transfection

Two siRNAs against human SRC (GenBank accession no. NM_005417) were designed as described previously (Reynolds *et al.,* 2004; Ui‐Tei *et al.,* 2004). The 19 nucleotide target sequences (SRC‐siRNA1: position 1489–1507 and SRC‐siRNA2: position 1684–1702) were synthesized into 64–65 mer oligonucleotides with BamHI/HindIII overhangs (Sigma Aldrich) and cloned into the expression vector pSilencer 3.0‐H1, containing pmaxGFP (Ambion Inc.). All clones were purified using an EndoFree Plasmid Maxi Kit (Qiagen Ltd) and sequenced (Geneservice Ltd). hASMC were transfected using the Basic Nucleofector® Kit and nucleofector device (Amaxa Biosystems). After 72 h, the transfection efficiency was >90%, confirmed by fluorescence microscopy.

### Protein lysate preparation and western blot

Cultured hASMCs were treated in serum‐free DMEM at 37°C. Preliminary studies showed that phosphorylation responses, although sustained for at least 5 min, peaked at ~30 s, so all subsequent acute treatments were for 30 s. Cells were immediately washed twice with ice‐cold PBS, followed immediately by application of cell lysis buffer (NEB) containing 1% phosphatase inhibitor cocktails 2 and 3 and 1% protease inhibitor cocktail (all Sigma). Cells were scraped into a tube and agitated before being placed on ice. Rat trachealis muscle was dissected free of adjoining cartilage, and epithelium was removed by scraping. Acute treatments were conducted in PSS/5% CO_2_, at 37°C, before the tissue was snap frozen in liquid nitrogen, pulverized and lysed in cell lysis buffer. All lysates were centrifuged at 9.2x *g*, and the supernatants were stored at −80°C.

Samples were boiled in NuPAGE LDS Sample Buffer (Invitrogen) at 95°C for 5 min before being loaded onto 4–12% NuPAGE Bis‐Tris gels (Invitrogen) for SDS‐PAGE. Sample protein content was determined using the bicinchoninic acid assay, calibrated against BSA protein standards, to enable loading of ~20 µg of protein per lane. Gels were run at 180 V for 1 h using an Xcell SureLock Mini‐Cell (Invitrogen) and MOPS running buffer (Invitrogen). Protein was transferred to a nitrocellulose membrane (Amersham) in 25 mM Tris, 192 mM glycine and 20% methanol, at 35 V for 1 h.

Membranes were blocked with 5% skimmed milk in Tris buffered saline (TBS) for 1 h at room temperature, followed by incubation with specific anti‐phospho‐protein primary antibody (typically 1:1000 dilution) in TBS with 1% skimmed milk and 0.1% Tween‐20 (TBS‐T), overnight at 4°C. Following washes in TBS‐T, HRP‐conjugated secondary antibody (typically 1:5000 dilution) was applied for 1 h at room temperature, followed by a final wash in TBS‐T. ‘Phospho’ proteins were visualized with Super‐Signal West Femto chemi‐luminescent Substrate (Thermo scientific). Membranes were then stripped in Restore western blot stripping buffer (Thermo Scientific), re‐blocked and re‐incubated with corresponding ‘total’ antibody and appropriate secondary antibodies, as above. ‘Total’ proteins were visualized with either ECL plus or ECL prime (Amersham, GE healthcare). Images were captured and quantified using the ChemiDoc XRS+ gel‐imaging system (Biorad). An estimate of the proportion of target protein that was phosphorylated was calculated as a ratio of ‘phospho’ over ‘total’ signal for each protein band from each gel, and the effects of acute treatments on these ratios was expressed as a percentage of control (untreated samples run on the same gel).

### [Ca^2+^]_i_ measurement

Cultured hASMCs were grown on glass cover‐slips until 70% confluent, followed by 7 days of serum starvation. Cells were loaded with 1 μM Fura PE‐3/AM in HBSS (containing in mM: 0.49 MgCl, 0.41 MgSO_4_, 4 KCl, 0.44 KH_2_PO_4_, 4.2 NaHCO_3_, 120 NaCl, 0.34 Na_2_HPO_4_, 20 HEPES and 2 CaCl) at room temperature for 40 min. Coverslips were mounted on an upright microscope and cells perfused with HBSS, containing test reagents as required. Changes in [Ca^2+^]_i_ were measured as a ratio of 340 nm over 380 nm emission intensities with a ×20 oil immersion UV objective and a microspectrofluorimeter (CairnResearch Ltd., U.K.). For each coverslip, ratios obtained in zero [Ca^2+^]_o_ and the absence of drug were taken as background fluorescence (auto‐fluorescence + residual basal [Ca^2+^]_i_) and subtracted from all subsequent measurements.

### Materials and reagents

Antibodies were obtained from cell signalling (anti‐phospho‐Src (tyr416); anti‐Src; anti‐phospho‐FAK (Y397); anti‐phospho‐FAK (Y576/577); anti‐FAK; anti‐phospho‐MLC (S19); anti‐MYPT1; anti‐MLC), Millipore (anti‐phospho‐MYPT1 (T696)), Sigma (anti‐rabbit IgG; anti‐mouse IgG). Kinase inhibitors were obtained from Sigma ((1R, 4r)‐4((R)‐1‐aminoethyl)‐*N*‐(pyridine‐4‐yl) cyclohexane carboxamide (Y27632); 6‐[4‐(3‐methanesulfonyl‐benzylamino)‐5‐trifluoromethyl‐pyrimidin‐2‐ylamino]‐3,4‐dihydro‐1H‐quinolin‐2‐one (PF‐573228); *N*‐[2‐[[[2‐[(2,3‐dihydro‐2‐oxo‐1H‐indol‐5‐yl)amino]‐5‐(trifluoromethyl)‐4‐pyrimidinyl]amino]methyl]phenyl]‐*N*‐methyl‐methanesulfonamide hydrate (PF‐431396) or Calbiochem: (4‐amino‐5‐(4‐chlorophenyl)‐7‐(dimethylethyl) pyrazolo[3,4‐d]pyrimidine (PP2); 4‐amino‐7‐phenylpyrazol[3,4‐d]pyrimidine (PP3). Cell culture and western blot materials were obtained from Cell Signalling, Invitrogen, GE Healthcare or Thermo Scientific. Nifedipine, YM58483, and cyclopiazonic acid and α‐haemolysin were from Sigma.

### Data analysis and statistics

All values are expressed as mean ± SEM. Non‐linear regression curve fitting was performed with SigmaPlot 10. Carbachol (CCh) concentration‐response curves were fitted using the Hill equation for the calculation of PD_2_ (‐LogM EC_50_) and maximum response (Max). Bradykinin concentration responses were biphasic, and were best fitted using a two‐site saturation model, for the characterization of a high affinity component (PD2‐1 and Max‐1) and a low affinity component (PD2‐2 and Max‐2). Statistical analysis of data was by Student's paired or un‐paired *t*‐test (two groups of data, single factor), one‐way ANOVA (more than two groups of data, single factor) or two‐way ANOVA (more than two groups of data, two factors), with Holm–Sidak *post* tests where appropriate, and as indicated in figure or table legends, using SigmaPlot 10. Differences were considered significant if *P* < 0.05.

## Results

#### GPCR‐mediated contraction of rat bronchioles is dependent on SrcFK, Rho‐kinase and FAK

**Figure 1:**
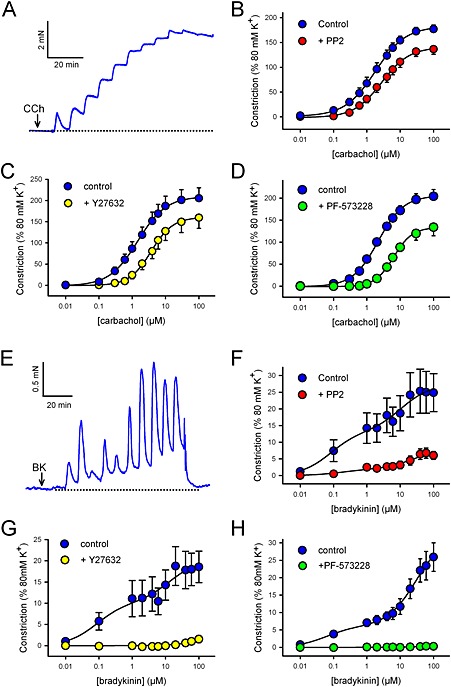
Effects of kinase inhibitors on carbachol and bradykinin‐induced contraction in rat bronchioles. Measurement of isometric tension in freshly isolated rat bronchioles. CCh (A–D) or BK (E–H) was applied cumulatively (0.01–100μM) at 5 min intervals. Representative traces show typical cumulative contractile responses to CCh (A) and BK (E). Arrows indicate the points where the first concentration was applied. Two responses were performed in each bronchiole, the second after application of the Src inhibitor PP2 (30 μM, 10 min, B: CCh, *n* = 8 or F: BK, *n* = 11), the Rho‐kinase inhibitor Y27632 (10 μM, 10 min, C: CCh, *n* = 6 or **G**: BK, *n* = 4), the FAK inhibitor PF‐573228 (10 μM, 10 min, D: CCh, *n* = 8 or H: BK, *n* = 5) or no inhibitor (not shown). Measurements were taken at the end of each 5 min exposure and data fitted by nonlinear regression. Data expressed as a % of that induced by 80 mM KPSS (mean ± SEM); see main text or Supporting Information Fig. S4 for effects on PD_2_ and max values.

We examined the contractile responses to CCh and bradykinin (BK) in rat bronchioles, whereby the bronchoconstrictors were applied cumulatively at 5 min intervals. Two concentration‐response curves were performed in each bronchiole (0.01–100μM), the first acting as a control and the second after pre‐incubation with either the SrcFK inhibitor PP2 (30 μM), the Rho‐kinase inhibitor Y27632 (10 μM), the FAK inhibitor PF‐573228 (10 μM) or no inhibitor (control). In addition, to account for possible off‐target effects of PP2 and PF‐573228, key contractile responses were also repeated with PP3 (30 μM), the negative control for PP2 and a dual FAK/PYK2 inhibitor, PF‐431396 (10 μM). CCh caused a sustained contraction at each dose (Figure 1A). The maximum response to CCh was significantly reduced by PP2 (*P* < 0.01, paired *t*‐test, *n* = 8), Y27632 (*P* < 0.01, paired *t*‐test, *n* = 6) and PF‐573228 (*P* < 0.05, paired *t*‐test, *n* = 8), and the PD_2_ was significantly increased by PP2 (−5.55 ± 0.09 vs. control −5.8 ± 0.14, *P* < 0.05, paired *t*‐test, *n* = 8), Y27632 (−5.4 ± 0.07 vs. control −5.82 ± 0.07, *P* < 0.01, paired *t*‐test, *n* = 6) and PF‐573228 (−5.21 ± 0.08 vs. control −5.69 ± 0.07, *P* < 0.001, paired *t*‐test, *n* = 8) (Figure 1A–D). PP3 had no significant effect on either PD_2_ (−5.6 ± 0.05 vs. control −5.72 ± 0.08, *n* = 7) or maximum contraction (144 ± 5% vs. control 149 ± 3%, *n* = 7). Conversely, PF‐431396 had similar effects as those of PF‐573228, causing a similar increase in PD_2_ (−5.20 ± 0.05 vs. control −5.90 ± 0.07, *P* < 0.001, paired *t*‐test, *n* = 7) and a similar reduction in maximum contraction (139 ± 6.3% vs. control 176 ± 9.3%, *P* < 0.001, paired *t*‐test, *n* = 7) (Supporting Information Figs. S2A and S3A). In time‐matched control responses, repeated in the absence of inhibitor, the maximum contraction of the second response was slightly increased (first repeat: 203 ± 22% vs. second repeat 228 ± 25%, *P* < 0.01, paired *t*‐test, *n*=10), but there was no significant change in PD_2_ (first repeat: −5.67 ± 0.11 vs. second repeat −5.63 ± 0.09, *n* = 10).

Bradykinin caused a prominent transient contraction and a smaller sustained component at each dose (Figure 1E). The concentration‐dependence of these responses appeared biphasic. Contraction at all concentrations of BK was significantly inhibited by PP2 (Figure 1F), with maximum amplitudes of both high and low affinity components being reduced (Max‐1 = 1.66 ± 0.5% vs. control 12.1 ± 4.1%; Max‐2 = 6.81 ± 2.0% vs. control 24.4 ± 6.0%, *P* < 0.05, *n* = 11), while PD_2_‐1 and PD_2_‐2 were both unchanged (PD_2_‐1 = −6.7 ± 0.21 vs. control −7.08 ± 0.11; PD_2_‐2 = −4.85 ± 0.27 vs. control −4.67 ± 0.22, *n* = 11) (see Supporting Information Fig. S4 for all BK dose responses curve fit data). BK‐induced responses were nearly abolished by Y27632 (Figure 1G) and abolished by PF‐573228 (Figure 1H), rendering curve fitting impossible. In time‐matched control responses, repeated in the absence of inhibitor, there were no changes in either Max or PD_2_ values for either the high or the low affinity component (not shown).

#### SrcFKs mediate GPCR‐induced Ca^2+^‐sensitization and Rho‐kinase activation, but FAK does not

**Figure 2:**
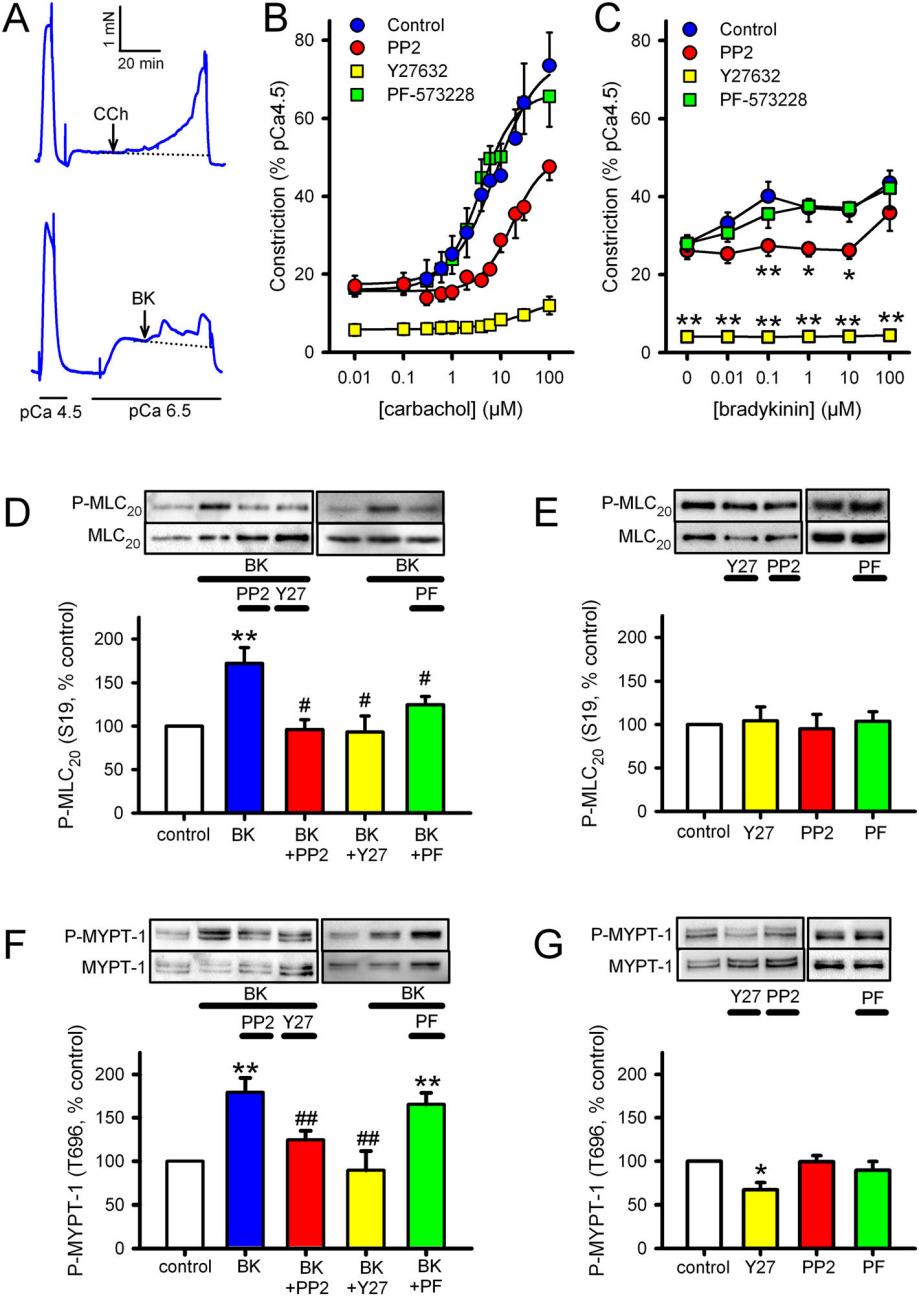
Effects of kinase inhibitors on contraction in α‐toxin permeabilized rat bronchioles and MLC_20_/MYPT‐1 phosphorylation in hASMC. (A–C) Measurement of isometric tension in α‐toxin permeabilized rat bronchioles. All responses were performed with pCa fixed at 6.5 and in the presence of 10 μM CPA and 1 μM GTP. (A) Representative traces showing CCh (upper panel) or BK (lower panel) being applied cumulatively (0.01–100 μM) at 5 min intervals, with arrows indicating where the first dose was applied. Responses were performed in the absence of inhibitor (control) or after pre‐incubation with either the Src inhibitor PP2 (30 μM, 10 min), the Rho‐kinase inhibitor Y27632 (10 μM, 10 min) or the FAK inhibitor PF‐573228 (10 μM, 10 min). Measurements were taken at the end of each 5 min exposure. Data are expressed as a % of that induced by pCa 4.5 (mean ± SEM). (B) CCh data were fitted by nonlinear regression (control, *n* = 10; PP2, *n* = 9; Y27632, *n* = 4; PF‐573228, *n* = 11); see main text for effects on CCh PD_2_. (C) BK data could not be fitted by nonlinear regression so were compared by two‐way RM ANOVA (control, *n* = 6; PP2, *n* = 6; Y27632, *n* = 4; PF‐573228, *n* = 6; *P < 0.05, **P < 0.01 vs. control). (D–G) Measurement of phosphorylation of MLC_20_ at S19 (P‐MLC_20_, D, E) and MYPT‐1 at T696 (P‐MYPT‐1, F, G), in hASMC. Representative blots show effects of treatment on ‘phospho’ and ‘total’ immunoreactivity for each protein. Bar charts show the effects of treatments on the degree of phosphorylation (mean ± SEM), expressed as a % of values from untreated (control) samples run on the same gels. (D) Effects of BK (1 μM 30 s) on MLC_20_ phosphorylation in the absence of inhibitor (*n* = 16) or after pretreatment with either PP2 (30 μM, 10 min, *n* = 11), Y27632 (10 μM, 10 min, *n* = 9) or PF‐573228 (PF, 10 μM, 10 min, *n* = 11). (E) Effects of inhibitors on basal MLC_20_ phosphorylation (*n* = 4–11). (F) Effects of BK (1 μM, 30 s) on MYPT‐1 phosphorylation in the absence of inhibitor (*n* = 13), or after pre‐application of PP2 (30 μM, 10 min, *n* = 13), Y27632 (10 μM, 10 min, *n* = 13), or PF‐573228 (10 μM, 10 min, *n* = 8). (G) Effects of inhibitors on basal MYPT‐1 phosphorylation (*n* = 4–11). Comparisons were by one‐way ANOVA with Holm–Sidak *post* tests: *P < 0.05 and **P < 0.01 versus control; #*P* < 0.5 and ##*P* <0.01 versus BK alone.

To clarify whether the effects of kinase inhibitors on bronchoconstrictor‐induced contraction is mediated through a Rho‐kinase‐dependent Ca^2+^‐sensitization pathway, CCh or BK concentration‐response curves were repeated in α‐toxin‐permeabilized rat bronchioles, with [Ca^2+^]_i_ fixed at pCa 6.5, in the absence or presence of PP2, Y27632 or PF‐573228. After α‐toxin‐permeabilization, bronchoconstictor concentration‐response curves were not repeatable (not shown), so controls and effects of antagonists were compared in separate bronchioles. CCh‐induced contraction was almost absent in the presence of Y27632 and was significantly smaller in the presence of PP2 (*P* < 0.05, unpaired *t*‐test, *n* = 9), but in the presence of PF‐573228 was not different from controls (Figure 2B). The PD_2_ was significantly greater after PP2 (−4.64 ± 0.13, vs. control ‐5.08 ± 0.12, *P* < 0.05, unpaired *t*‐test, *n* = 9), but was no different in PF‐573228. The underlying pCa 6.5 contraction was unaffected by either PP2 or PF‐573228, but was partially inhibited by Y27632 (61 ± 8% block, *P* < 0.05 vs. absence of Y27632, paired *t*‐test, *n* = 6). BK also produced a modest (relative to CCh) concentration‐dependent contraction in permeabilized bronchioles (Figure 2A and C). The concentration‐dependence of these responses again appeared biphasic, but their small amplitude and poor sustainability rendered curve‐fitting impossible. Nevertheless, peak responses were significantly smaller or absent in the presence of PP2 or Y27632, respectively, and no different in PF‐573228 (Figure 2C).

To confirm that Rho‐kinase is being activated by 30 s exposure to BK (1 μM) or CCh (100 μM), and whether this activation relates to subsequent activation of MLCK, we measured phosphorylation of MYPT‐1 at T696, a known phosphorylation target of Rho‐kinase (Feng *et al.,* 1999), and of MLC_20_ at S19, the main target of MLCK. Furthermore, in order to reveal a possible interaction between Rho‐kinase and SrcFK or FAK with relation to MLC_20_ phosphorylation, we examined the effects of inhibitors of Rho‐kinase, SrcFK or FAK on both phosphorylation responses in hASMC. Phosphorylation of MLC_20_ and MYPT‐1 were both significantly enhanced by BK. This enhancement was significantly reduced by PP2 and abolished by Y27632 at both sites, while PF‐573228 significantly reduced the enhancement of MLC_20_, but not MYPT‐1 phosphorylation (Figure 2D and F). CCh also enhanced phosphorylation of both proteins, but to a lesser extent than BK. Basal MLC_20_ phosphorylation was insensitive to all three inhibitors (Figure 2E), while basal MYPT‐1 phosphorylation was partially sensitive to Y27632, but insensitive to PP2 or PF‐573228 (Figure 2G).

#### Bronchoconstrictors enhance SrcFK and FAK auto‐phosphorylation

**Figure 3:**
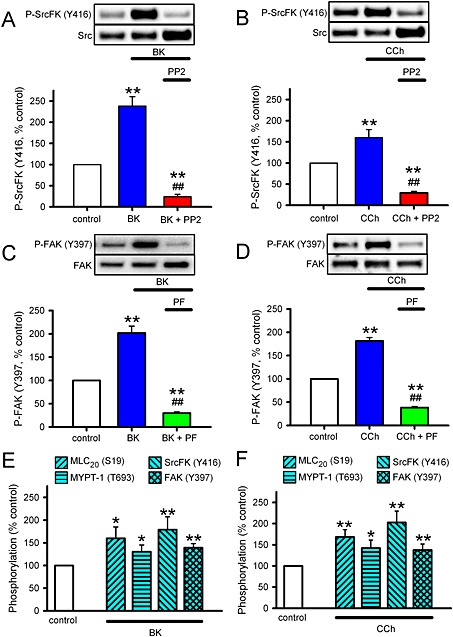
Effects of broncoconstrictors on SrcFK and FAK auto‐phosphorylation in hASMC and rat trachealis. (A–D) Measurements of auto‐phosphorylation of SrcFK at Y416 (P‐SrcFK, A, B) and auto‐phosphorylation of FAK at Y397 (P‐FAK, C, D) in hASMC. Representative blots show effects of treatments on ‘phospho’ and ‘total’ immunoreactivity for each protein. Bar charts show the effects of treatments on the degree of phosphorylation (mean ± SEM), expressed as a % of values from untreated (control) samples run on the same gels. (A) Effect of BK (1 μM, 30 s) in the absence (*n* = 12) or presence of PP2 (30 μM, 10 min, *n* = 7) on P‐SrcFK (Y416). (B) Effect of CCh (100 μM, 30 s) in the absence (*n* = 13) or presence of PP2 (*n* = 8) on P‐SrcFK (Y416). (C) Effect of BK in the absence (*n* = 15) or presence of PF‐573228 (PF, 10 μM, 10 min, *n* = 13) on P‐FAK (Y397). (D) Effect of CCh in the absence (*n* = 16) or presence of PF‐573228 (*n* = 11) on P‐FAK (Y397). Comparisons by one‐way ANOVA with Holm–Sidak *post* tests: **P < 0.01 versus control; ##*P* < 0.01 versus BK or CCh alone. (E, F) Measurements of phosphorylation of MLC_20_ (S19), MYPT‐1 (T696), SrcFK (Y416) and FAK (Y397) in rat trachealis muscle. (E) Effects of BK (1 μM, 30 s, *n* = 8). (F) Effects of CCh (100 μM, 30 s, *n* = 8). Comparisons by unpaired *t*‐test: *P<0.05, **P<0.01 versus control.

In order to confirm that the influence of SrcFK and FAK on contraction and Rho‐kinase activity occurs in direct response to bronchoconstrictor stimulation, we also examined the effects of BK or CCh on SrcFK auto‐phosphorylation at Y416 and FAK auto‐phosphorylation at Y397, as a reflection of respective changes in kinase activity (Calalb *et al.,* 1995; Xu *et al.,* 1999). In hASMC, auto‐phosphorylation of both kinases was significantly enhanced by both agents (Figure 3 A–D). As expected, SrcFK phosphorylation was almost abolished by PP2, and FAK phosphorylation was almost abolished by PF‐573228, both confirming the selectivity of the phospho‐antibodies and validating the choice of kinase inhibitor concentrations used. In rat trachealis muscle, SrcFK and FAK auto‐phosphorylation were also enhanced by BK and CCh, as was phosphorylation of MLC_20_ (S19) and MYPT‐1 (Y397), (Figure 3E, F). Bronchoconstrictor‐induced FAK Y397 phosphorylation was noticeably weaker in rat trachealis than in hASMC.

#### FAK and SrcFK influence SOCE/ROCE and VOCE

**Figure 4:**
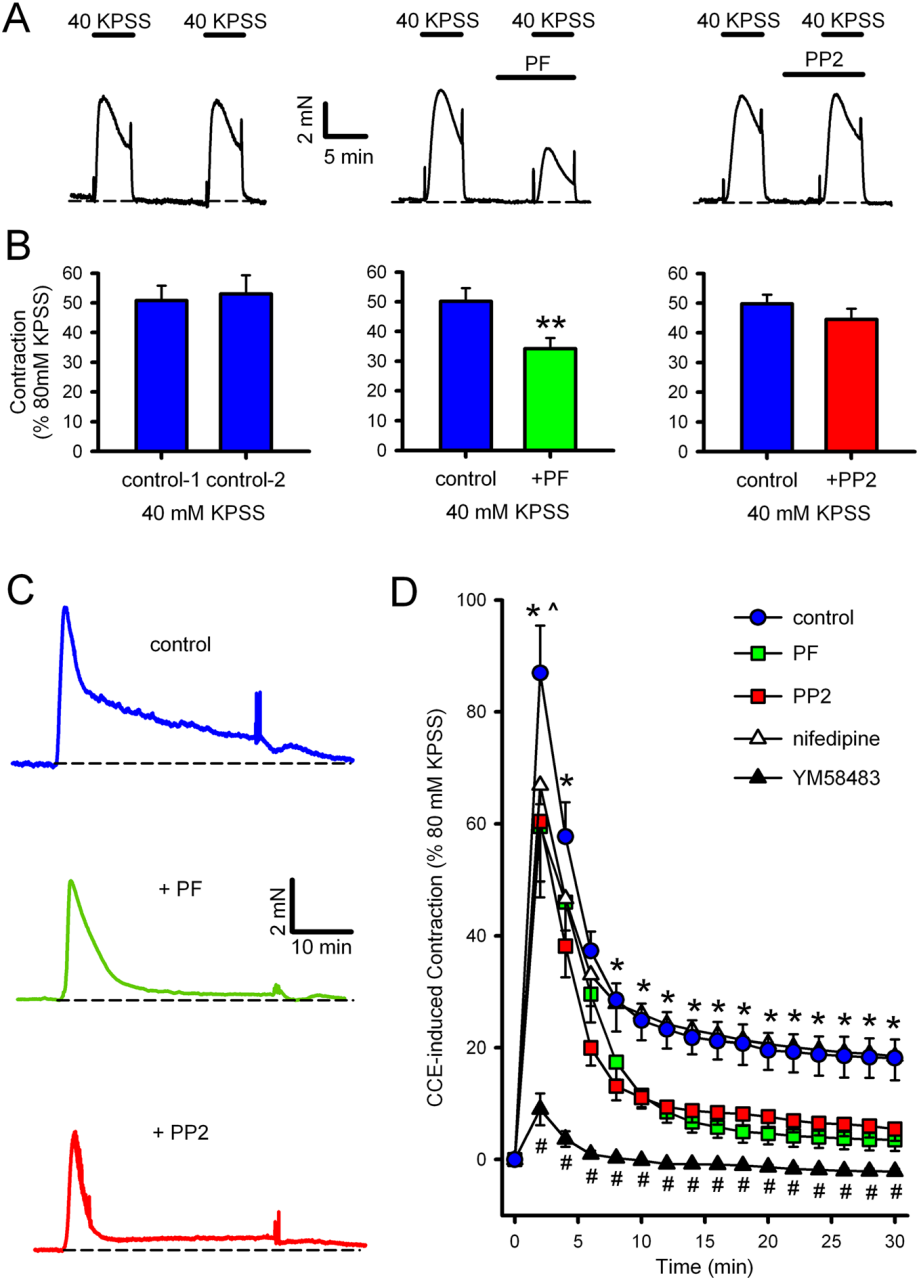
Effects of SrcFK and FAK inhibitors on VOCE‐ or SOCE‐associated contraction in rat bronchioles. (A, B) VOCE‐associated contractions induced by 40 mM KPSS. Representative traces (A) and mean measurements of peak amplitude (B, ± SEM), showing effects of two contractions in the absence of inhibitor (left panels, *n* = 8), the effect of PF‐573228 on the second contraction (PF, middle panels, 10 μM, 5 min pre‐incubation, *n* = 7) or the effect of PP2 on the second contraction (30 μM, right panels, 5 min pre‐incubation, *n* = 9). Comparisons by paired *t*‐test: **P < 0.01 versus control. (C, D) SOCE‐associated contraction induced by 10 μM CPA/200 μM EGTA/zero Ca^2+^, followed by re‐application of 2 mM Ca^2+^. Representative traces (C) and mean measurements of amplitude of contractile responses at 2 min intervals after re‐application of Ca^2+^ (D, ± SEM), showing control response (*n* = 14) and effects of pre‐incubation with PF‐573228 (*n* = 9), PP2 (*n* = 9), nifedipine (*n* = 10) or YM58483 (*n* = 5). Comparisons by two‐way ANOVA with Holm–Sidak *post* tests: *P < 0.05 for control versus PF‐573228 or PP2. #*P*<0.01 for control versus YM58483. ^*P* < 0.05 for control versus nifedipine at 2 min only.

We examined the effects of PP2 and PF‐753228 on VOCE‐mediated contraction by sub‐maximal depolarization with 40 mM KPSS. In control experiments, contraction amplitude induced by two consecutive KPSS exposures was not significantly different, while when PP2 (30 μM) or PF‐573228 (10 μM) was applied between the first and second exposures, the contractile response was modestly but significantly reduced by PF‐573228, but not by PP2 (Figure 4A and B). To rule out a direct Ca^2+^‐channel antagonist effect of PF‐573228, we also examined its effect on contraction induced by maximal depolarization with 80 mM KPSS. This contraction was significantly less sensitive to PF‐573228 than the 40 mM KPSS contraction (11.5 ± 2.7% inhibition, *n* = 6, vs. 30.2 ± 6.3% inhibition of 40 mM KPSS, *n* = 7; *P <* 0.05 by unpaired *t*‐test). We then examined the effects of PP2 and PF‐573228 on SOCE‐mediated contraction. SR Ca^2+^ was first depleted with cyclopiazonic acid (CPA, 10 μM) in the absence of extracellular Ca^2+^ and presence of 200 μM EGTA, then 2 mM Ca^2+^ was re‐applied. CPA was used here instead of a GPCR agonist because it would have been difficult to separate effects of the agonist on Ca^2+^ entry from those on Rho‐kinase‐mediated Ca^2+^ sensitization. In control experiments, re‐application of 2 mM Ca^2+^ induced a biphasic contraction, which peaked at ~2 min and slowly decayed to ~20% of 80 mM KPSS after 30 min. In the presence of either PP2 or PF‐573228, the sustained component was significantly smaller, decaying to <10% of 80 mM KPSS after 30 min (Figure 4C and D). PP3 was without significant effect on this response, ruling out the possibility of a non‐specific SOCE blocking effect of PP2 (Supporting Information Fig. S2B), while PF‐431396 inhibited the response in a near‐identical way to that of PF‐573228, supporting a specific role for FAK in this response (Supporting Information Fig. S3B). To see whether these effects of PP2 and PF‐573228 were due to an effect of SrcFK or FAK on SOCE itself or on secondary activation of VOCE, the effect of the SOCE blocker YM58483 (10 μM) and the Ca^2+^ channel antagonist nifedipine (2 μM) on the SOCE‐mediated contraction was also determined. Apart from a small residual transient contraction, the response was abolished by YM58483, while nifedipine was without effect, apart from a small reduction in the peak response (Figure 4D).

**Figure 5:**
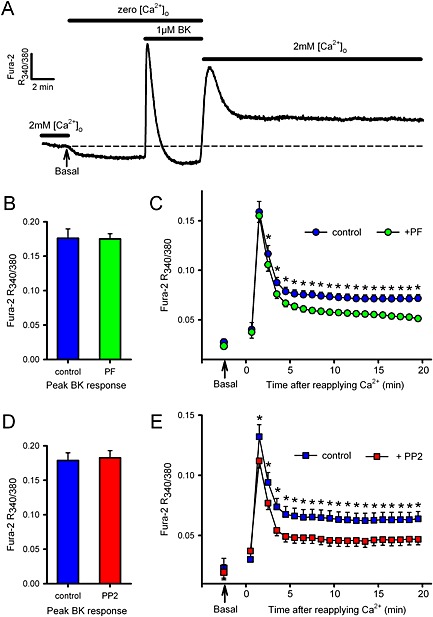
Effects of SrcFK and FAK inhibitors on SOCE/ROCE‐associated [Ca^2+^]_i_ responses in hASMC. A: Representative control trace of [Ca^2+^]_i_ in Fura‐2 loaded hASMC, as determined by the ratio of fluorescence at 340 nm/380 nm. Arrow indicates the point at which pre‐stimulus basal [Ca^2+^]_i_ was recorded in 2 mM [Ca^2+^]_o_ (and extrapolated by dashed line). The buffer was then switched to zero [Ca^2+^]_o_ until a new baseline was established, and 1 μM BK added for 5 min. Finally, 2 mM [Ca^2+^]_o_ was reapplied for 20 min. Responses were performed either in the absence or presence of FAK inhibitor PF‐573228 (B, C: PF, 10 μM, added 5 min prior to BK, *n* = 15 vs. 15 matched controls) or SrcFK inhibitor PP2 (D, E: 30 μM, added 5 min prior to BK, *n* = 10 vs. eight matched controls). Measurements were made of the peak BK‐induced transient (B, D: arbitrary units, mean ± SEM) and of the response to reapplication of 2 mM [Ca^2+^]_o_ (C, E: arbitrary units, measured at 1 min intervals, mean ± SEM), compared with the pre‐stimulus basal [Ca^2+^]_i_ in 2 mM [Ca^2+^]_o_ (indicated by arrows). Background fluorescence (in zero [Ca^2+^]_o_, prior to the application of BK) was subtracted from all other measurements. Comparisons by un‐paired *t*‐test (B, D) or two‐way ANOVA with Holm–Sidak post tests (C, E: *P < 0.05 vs. matched controls).

To support contraction data and to further eliminate the possibility that SrcFK and FAK were indirectly influencing SOCE via an action on SR Ca^2+^ release, we also examine the effects of PP2 and PF‐753228 on SOCE [Ca^2+^]_i_ responses in Fura‐2 loaded hASMC, using BK as the initial SR‐emptying stimulus. After the recording of an initial baseline in 2 mM Ca^2+^, and then for 5 min in nominally Ca^2+^‐free buffer, the addition of BK (1 μM) caused a near instantaneous increase in [Ca^2+^]_i_. This decayed back to the baseline within ~2 min; a response typical of GPCR‐induced SR Ca^2+^‐release. 2 mM Ca^2+^ was then re‐applied for 20 min, with simultaneous washout of BK. This induced a biphasic rise in [Ca^2+^]_i_ with a similar time course to the SOCE contractile responses. In the presence of either PP2 or PF‐573228, the sustained component was reduced by ~50%, while the initial transient component and the initial BK‐induced SR release were both unaffected (Figure 5).

#### Interaction between SrcFK and FAK

**Figure 6:**
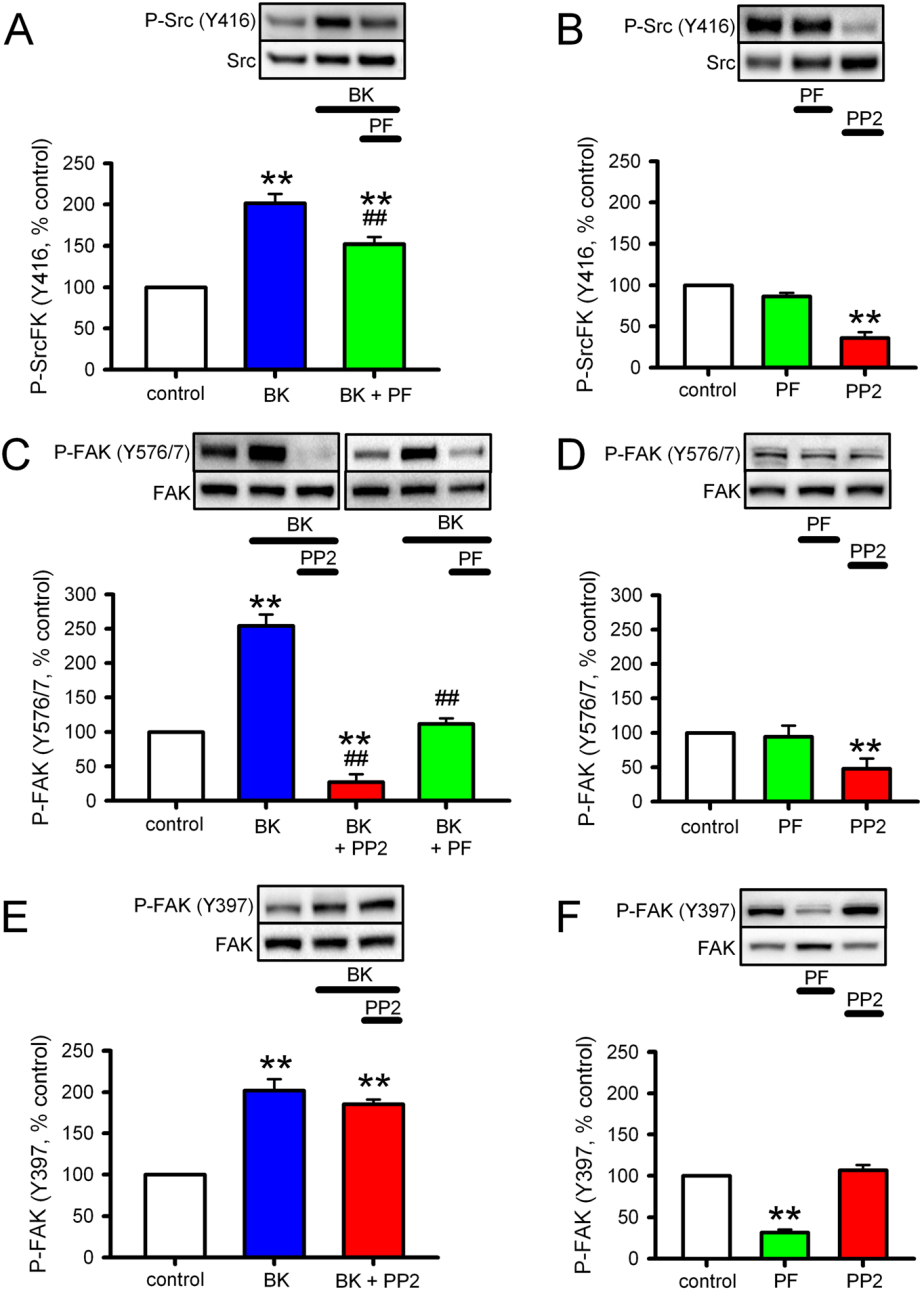
Interaction between SrcFK and FAK in response to BK in hASMCs. Measurements of auto‐phosphorylation of SrcFK at Y416 (P‐SrcFK, A, B), phosphorylation of FAK at the Y576/577 dual site (C, D), and auto‐phosphorylation of FAK at Y397 (P‐FAK, E, F) in hASMC. Representative blots show effects of treatments on ‘phospho’ and ‘total’ immunoreactivity for each protein. Bar charts show the effects of treatments on the degree of phosphorylation (mean ± SEM), expressed as a % of values from untreated (control) samples run on the same gels. (A) Effects of BK (1μM, 30 s) on P‐SrcFK (Y416) in the absence (*n* = 14) or presence of the FAK inhibitor PF‐573228 (PF, 10μM 10 min, *n* = 7). (B) Effects of PF (*n* = 4) or the SrcFK inhibitor PP2 (30μM, 10 min, *n* = 4) on basal P‐SrcFK (Y416). (C) Effects of BK on P‐FAK (Y576/577) in the absence (*n* = 11) or presence of PP2 (*n* = 6) or PF (*n* = 7). (D) Effects of PF (*n* = 4) or PP2 (*n* = 5) on basal P‐FAK (Y576/577). (E) Effect of BK on P‐FAK (Y397) in the absence (*n* = 14) or presence of PP2 (*n* = 4). (F) Effects of PF (*n* = 4) or PP2 (*n* = 4) on basal P‐FAK (Y397). Comparisons by one‐way ANOVA with Holm–Sidak *post* tests: **P < 0.01 versus control or ##*P* < 0.01 versus BK alone.

Because SrcFK and FAK appear to be sharing some but not all of the contraction signalling pathways investigated in this study, and in the light of previous evidence suggesting cooperation between the two kinases, we investigated the influence of FAK on SrcFK auto‐phosphorylation and *vice versa*. Enhancement of SrcFK (Y416) auto‐phosphorylation by BK was inhibited by PF‐573228 by ~50% (Figure 6A), while basally, this phosphorylation was insensitive to PF‐573228 but inhibited by PP2 (Figure 6B). FAK kinase activity is also reportedly influenced by Src‐dependent phosphorylation on FAK Y576/577 (Calalb *et al.,* 1995). Phosphorylation at this dual site was enhanced by BK, and this enhancement was nearly abolished by PP2 and partially inhibited by PF‐573228 (Figure 6C), while basally, this phosphorylation was inhibited by PP2, but not PF‐573228 (Figure 6D). However, BK‐induced enhancement of FAK (Y397) auto‐phosphorylation was unaffected by PP2 (Figure 6E). Basal phosphorylation of FAK (Y397) was also insensitive to PP2, but was inhibited by PF‐53228 (Figure 6F).

#### c‐Src is the principle SrcFK mediating bronchoconstrictor‐induced phosphorylation responses

**Figure 7:**
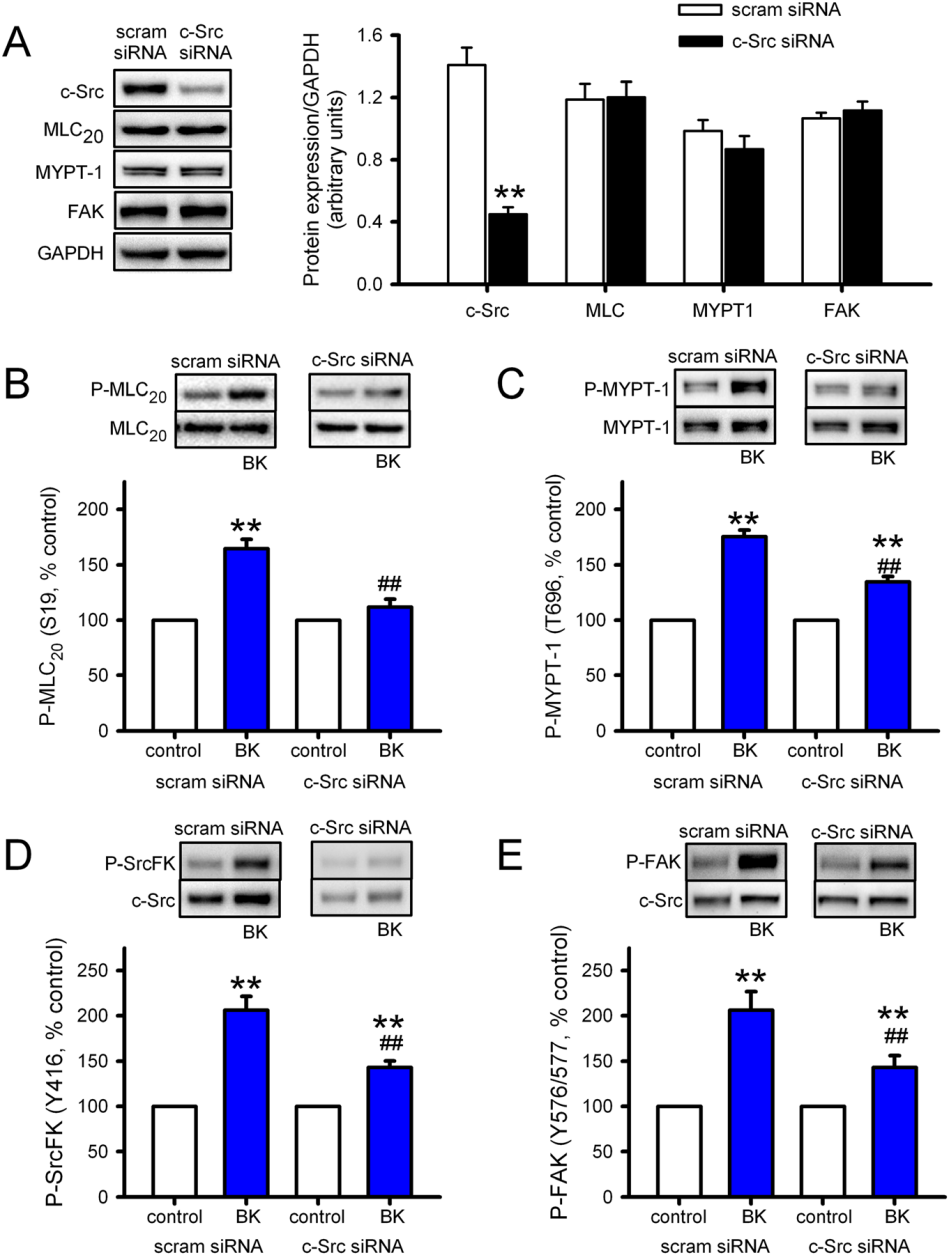
Effects of c‐Src siRNA on bronchoconstrictor‐induced MLC_20_, MYPT‐1, SrcFK and FAK phosphorylation in hASMCs. Measurement of protein expression and phosphorylation responses to BK (1 μM, 30 s) in hASMCs after transfection with c‐Src siRNA or scrambled siRNA (scram). (A) Effect of c‐Src siRNA on c‐Src protein expression and (lack of) effect on MLC_20_, MYPT‐1 and FAK expression. Data normalized to GAPDH expression in the same samples (arbitrary units, *n* = 8–12). Comparisons by unpaired *t*‐test: **P < 0.01 versus scram siRNA. (B) Effect of BK on MLC_20_ phosphorylation at S19 (P‐MLC_20_) after transfection with scram siRNA (*n* =16) or c‐Src siRNA (*n* = 16). (C) Effect of BK on MYPT‐1 phosphorylation at T696 (P‐MYPT‐1) after transfection with scram siRNA (*n* = 14) or c‐Src siRNA (*n* = 14). (D) Effect of BK on SrcFK phosphorylation at Y416 (P‐SrcFK) after transfection with scram siRNA (*n* = 16) or c‐Src siRNA (*n* = 16). (E) Effect of BK on FAK phosphorylation at Y576/577 (P‐FAK) after transfection with scram siRNA (*n* = 10) or c‐Src siRNA (*n* = 10). Comparisons by two‐way ANOVA: **P < 0.01 versus control; ##*P* < 0.01 versus scram siRNA.

Multiple members of the Src‐family of kinases are expressed in ASM, including c‐Src, Fyn, Yes and Lyn (Sakai *et al.,* 2010). For this reason, and the fact that the phospho‐SrcFK antibody cannot distinguish between Src family members, we re‐examined the effects of acute BK treatment on MLC_20_ (S19), MYPT‐1 (T696), SrcFK (Y416) and FAK (Y576/577) phosphorylation in hASMC after transfection with c‐Src siRNA or scrambled siRNA. Specificity of c‐Src siRNA was verified by a ~70% reduction in c‐Src protein expression, while expressions of MLC_20_, MYPT‐1 and FAK were all unaffected (Figure 7A). The scrambled siRNA had no effect on any of the proteins examined. c‐Src siRNA inhibited BK‐induced phosphorylation of MLC_20_ by ~80%, while responses of MYPT‐1 (T696), SrcFK (Y416) and FAK (Y576/577) to BK were all inhibited by ~60%, compared with matched scrambled siRNA transfected cells (Figure 7 B–D).

## Discussion

We examined the role of SrcFK and FAK kinase activity in bronchoconstrictor‐induced contraction of rat‐isolated bronchioles and in [Ca^2+^]_i_ and phosphorylation responses in hASMC. Contraction was induced and MLC_20_ phosphorylation was enhanced by the bronchoconstrictors BK and CCh, and these responses were sensitive to inhibition of both SrcFK and FAK. Using auto‐phosphorylation as an indication of kinase activity, both SrcFK and FAK were activated by both agents in hASMC and rat trachealis, suggesting an important role for these kinases in GPCR‐induced ASM contraction in both humans and rodents. PP3, the negative control for PP2, was without effect on contraction, while PF‐431396, another inhibitor of FAK, had similar effects as PF‐573228, reducing the likelihood of our results being influenced by non‐specific effects of PP2 or PF‐573228. Amongst the several Src family members, both c‐Src and Fyn have been implicated in vascular smooth muscle contraction (Nakao *et al.,* 2002; Knock *et al.,* 2008), and Lyn is activated by muscarinic agonists in ASM (Pertel *et al.,* 2006). Here however, the effects of c‐Src siRNA suggest that 60–80% of all the bronchoconstrictor‐induced phosphorylation responses investigated herein were specifically mediated by c‐Src.

To further characterize the signalling pathways through which SrcFK and FAK mediate ASM contraction, we first focussed on their role in Rho‐kinase dependent Ca^2+^‐sensitization, a process whereby inhibition of MLCP results in enhanced MLC_20_ phosphorylation, and hence force generation, without the requirement for an increase in [Ca^2+^]_i_ (Somlyo and Somlyo, 2003). Both BK and CCh‐induced contraction were highly sensitive to Rho‐kinase inhibition with Y27632. Furthermore, a component of the contractile response to both BK and CCh persisted when bronchioles were permeabilized with α‐toxin to prevent changes in intracellular Ca^2+^. We found that these contractile responses were dependent on Rho‐kinase and SrcFK, but not FAK. Furthermore, we found that MYPT‐1 phosphorylation on T696, an indicator of Rho‐kinase‐mediated MLCP inhibition (Feng *et al.,* 1999), is also enhanced by BK and CCh and that this enhancement is sensitive to inhibition of Rho‐kinase and SrcFK, but not of FAK. This influence of SrcFK on Rho‐kinase activity occurred specifically in response to agonist stimulation, because baseline phosphorylation of MYPT‐1 and MLC_20_ and baseline pCa6.5 contraction in permeabilized bronchioles were not affected by SrcFK inhibition. Clearly, these results indicate that SrcFK mediates GPCR‐induced smooth muscle contraction in part via activation of Rho‐kinase. Importantly, this is the first direct demonstration of an interaction between SrcFK and Rho‐kinase in ASM and in vessels of a size relevant to the control of airway resistance, consistent with the implied importance of Rho‐kinase in airway hyper‐responsiveness from studies in whole animal or isolated upper airways (Yoshii *et al.,* 1999; Schaafsma *et al.,* 2006). Interestingly, receptor TK stimulation also induces Rho‐kinase‐dependent ASM contraction (Gosens *et al.,* 2004), and other responses of ASM to growth factor stimulation are also SrcFK‐dependent (Krymskaya *et al.,* 2005). Thus SrcFK may be a point of convergence for the activation of Rho‐kinase in response to either GPCR or growth factors.

Rho‐kinase is directly activated by the small G‐protein RhoA, which is itself activated by guanine nucleotide exchange factors (RhoGEFs). RhoGEFs are known to be activated or modulated by various non‐receptor TKs (Chikumi *et al.,* 2002; Ying *et al.,* 2009; Guilluy *et al.,* 2010), in addition to G_12/13_ binding. It is therefore conceivable that SrcFK may be activating RhoA/Rho‐kinase via direct phosphorylation of a RhoGEF. Alternatively, they may do so via the prior activation of another kinase, such as FAK, PYK2 or JAK2 (Calalb *et al.,* 1995; Andreev *et al.,* 2001; Singh *et al.,* 2011). Our results are not inconsistent with this hypothesis, but exclude FAK as the intermediary kinase in this instance.

We next focussed on the role of SrcFK and FAK in Ca^2+^ signalling. Gq/PLC‐β‐coupled GPCRs induce Ca^2+^ entry through three main pathways: DAG‐sensitive TRP channel opening (ROCE), IP_3_‐dependent depletion of SR Ca^2+^ and subsequent STIM1/Orai1/TRP‐dependent influx (SOCE), and subsequent depolarization‐induced opening of l‐type Ca^2+^ channels (VOCE) (Kawasaki *et al.,* 2006; Wang *et al.,* 2008). Several members of the TRPC family of channels, in addition to being modulated by DAG, are subject to modulation by phosphorylation, and tyrosine phosphorylation of TRPC channels is SrcFK‐dependent, contributing to either ROCE or SOCE (Kawasaki *et al.,* 2006). Moreover, association of STIM1 with Orai1 in response to SR depletion, and subsequent Ca^2+^ influx, is partially dependent on SrcFK‐mediated phosphorylation of STIM1 (Lopez *et al.,* 2012). In accord with these previous studies, we found that CPA‐induced SOCE‐dependent contraction in rat bronchioles and BK‐induced Ca^2+^ influx in hASMC were both similarly sensitive to SrcFK inhibition. Interestingly, we found that these responses were also similarly sensitive to FAK inhibition. This, to our knowledge, is the first indication that FAK may be contributing to GPCR‐induced Ca^2+^ responses and contraction in human and rodent ASM, via upstream modulation of SOCE and/or ROCE. Some GPCR agonists, notably angiotensin II, also mediate SR Ca^2+^ release via SrcFK‐dependent tyrosine phosphorylation of PLC‐γ (Schmitz *et al.,* 1997). However, there is no indication that this is occurring in our study, because we found that neither PP2 nor PF‐573228 altered the BK‐induced Ca^2+^ transients indicative of SR release.

Our finding that the CPA‐induced SOCE contraction was minimally affected by the Ca^2+^ channel antagonist nifedipine suggests that VOCE secondary to SOCE‐induced depolarization was not contributing substantially to this response. However, VOCE may also be activated more directly via a number of signalling pathways including stretch‐activated phosphatidylcholine‐specific PLC‐derived DAG (Mauban *et al.,* 2015) or integrin‐directed tyrosine phosphorylation. Regarding the latter, engagement of integrin α5β1 induces SrcFK and FAK‐dependent phosphorylation of the α1c subunit of the l‐type Ca^2+^ channel in vascular smooth muscle, with the likeliest sequence of events being integrin‐induced FAK auto‐phosphorylation followed by SrcFK recruitment by FAK (Owen *et al.,* 1999; Salazar and Rozengurt, 2001) and subsequent direct phosphorylation of the channel by SrcFK (Wu *et al.,* 2001; Gui *et al.,* 2006). Similarly in ASM, VOCE may also be enhanced via stretch or adhesion‐induced FAK activity (Smith *et al.,* 1998; Tang *et al.,* 1999). In bronchioles, we found that contraction induced by depolarization with sub‐maximal (40 mM) K^+^ was sensitive to PF‐573228 but not PP2, which suggests that FAK may be activating VOCE independently of SrcFK. However, contraction induced by maximum depolarization with 80 mM K^+^ was considerably less sensitive to PF‐573228, ruling out a non‐specific effect of PF‐573228 on Ca^2+^ channel opening *per se*. We did not systematically examine the effects of stretch on contractile responses, as carried out previously in trachea (Tang *et al.,* 1999), but applied a standard degree of stretch to maximize active tension responses to bronchoconstrictors or KPSS. Similarly, adherent hASMC are assumed to be under a degree of self‐induced basal tension (Deguchi *et al.,* 2005). In light of this, it is worth noting that the relatively weak FAK auto‐phosphorylation observed in trachealis samples treated with BK or CCh, compared with similarly treated hASMC, may have been because no stretch was applied to trachealis samples during BK or CCh treatment prior to snap freezing for protein extraction.

**Figure 8:**
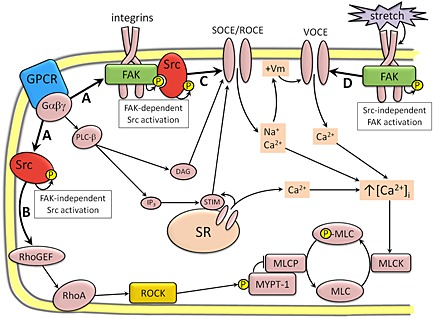
Proposed role for SrcFK and FAK in bronchoconstrictor or depolarization‐induced ASM contraction, based on the results of the current study and existing literature. (A) GPCR activate both SrcFK and FAK, presumably via interaction with heterotrimeric G‐protein sub‐units (e.g. Gα_q_, Gα_12/13_ or Gβγ) or downstream signalling molecules. Activated SrcFK forms two distinct sub‐populations: one FAK‐independent and one FAK‐dependent. (B) FAK‐independent SrcFK induces Rho‐kinase to phosphorylate MYPT‐1, thus enhancing myosin light‐chain phosphorylation (P‐MLC) through inhibition of myosin light‐chain phosphatase (MLCP). SrcFK are perhaps activating Rho‐kinase via the tyrosine phosphorylation of RhoA‐associated proteins such as RhoGEFs. (C) A FAK/Src complex may be mediating store‐operated and/or receptor‐operated Ca^2+^ entry (SOCE/ROCE) via the tyrosine phosphorylation of TRP channels, or associated signalling proteins, such as STIM1, in association with DAG. (D) FAK is also independently enhancing voltage‐operated Ca^2+^ entry (VOCE), perhaps via direct phosphorylation of voltage‐dependent Ca^2+^ channels, in response to the additional stimulus of stretch or cellular adhesion, via integrin engagement.

In smooth muscle contraction, it has been assumed that the mutual dependence between SrcFK and FAK relates primarily to the recruitment of contractile fibres through actin polymerization and focal attachment formation (Gerthoffer and Gunst, 2001; Tang *et al.,* 1999; Gunst *et al.,* 2003). It is therefore of note that we only see possible evidence of such mutuality with regard to SOCE/ROCE activity, but not in relation to Rho‐kinase activation (SrcFK only) or VOCE activity (FAK only). The selective sensitivity of basal SrcFK auto‐phosphorylation to PP2 and of basal FAK auto‐phosphorylation to PF‐537228 confirms the specificity of these two antagonists for their intended targets at the concentrations used in this study. Therefore, our finding that BK‐induced SrcFK auto‐phosphorylation is partially PF‐537228‐sensitive suggests a partial dependence of GPCR‐induced SrcFK activity on FAK. This is probably because SrcFK can be activated via disruption of intramolecular auto‐inhibition by association of the SH2 domain with the phosphorylated Y397 of FAK (Xing *et al.,* 1994). Conversely, we show that BK‐induced FAK auto‐phosphorylation is wholly independent of SrcFK activity. In response to adhesion, in non‐muscle cells, FAK auto‐phosphorylation is enhanced by SrcFK‐mediated phosphorylation on Y576/577 (Calalb *et al.,* 1995; Salazar and Rozengurt, 2001). However, despite the fact that we show that FAK Y576/577 phosphorylation is also induced by BK and almost entirely SrcFK‐mediated, this phosphorylation does not correlate with enhanced FAK auto‐phosphorylation. A similar discrepancy was observed in fibroblasts where adhesion‐induced FAK auto‐phosphorylation was SrcFK‐dependent but GPCR‐induced FAK auto‐phosphorylation was not, despite both stimuli inducing SrcFK‐dependent Y576/577 phosphorylation (Salazar and Rozengurt, 2001). Taken together, our results imply two things about these interactions in ASM: firstly, that GPCR can induce FAK activation without prior activation of SrcFK, and secondly, that there may be two sub‐populations of BK/CCh‐activated SrcFK; one FAK‐dependent, resulting in modulation of SOCE/ROCE, and the other FAK‐independent, resulting in activation of Rho‐kinase (as summarized in Figure 8).

In conclusion, our data suggest an important role for SrcFK and FAK in bronchoconstrictor‐mediated contraction in ASM, with the two kinases acting together to induce SOCE/ROCE, and independently to mediate Rho‐kinase‐dependent Ca^2+^ sensitization and VOCE respectively. These findings may inform the search for new drug targets for the treatment of obstructive lung diseases such as asthma, and in particular, support the suggested key role for SrcFK in experimental airway hyper‐responsiveness (Sakai *et al.,* 2010; Katsumoto *et al.,* 2013).

## Funding

Wellcome Trust: #087776. British Heart Foundation: FS/12/43/29608.

## Author contributions

All other authors reviewed the manuscript critically for important intellectual content. All authors agree to be accountable for all aspects of the work in ensuring that questions related to the accuracy or integrity of any part of the work are appropriately investigated and resolved.

## Conflict of interest

Authors declare that they have not any conflict of interest.

## Supporting information


**Figure S1** Identification of hASMC as smooth muscle by positive staining with anti‐smooth muscle‐actin (panel A), anti‐desmin (panel B) and anti‐calponin (panel C), visualised with Alexa Fluor®488 labelled secondary antibody (Lifetechnologies) and fluorescent microscopy. Cells were also stained with TRITC‐labelled phalloidin to confirm the presence of stress fibres in resting cells (Panel D). In Panel D, nuclei are visualised by staining with Hoechst. Scale bar =20μm.
**Figure S2** Effect of PP3 on contractile responses in rat bronchioles.
**Figure S3** Effect of PF‐431396 on contractile responses in rat bronchioles.
**Figure S4** Effects of SrcFK, Rho‐kinase and FAK inhibition on bradykinin‐induced contractile responses in rat bronchioles.

Supporting info item
