# Supplementary material for: Divergent modulation of Rho‐kinase and Ca2+ influx pathways by Src family kinases and focal adhesion kinase in airway smooth muscle
Source: Br J Pharmacol. 2015 Oct 23;172(22):5265–80. doi: 10.1111/bph.13313 (PMC4864488; doi:10.1111/bph.13313)
Supplement: Supplementary file 1 — Supporting info item [file BPH-172-5265-s001.docx]

**Supplementary Figure 1
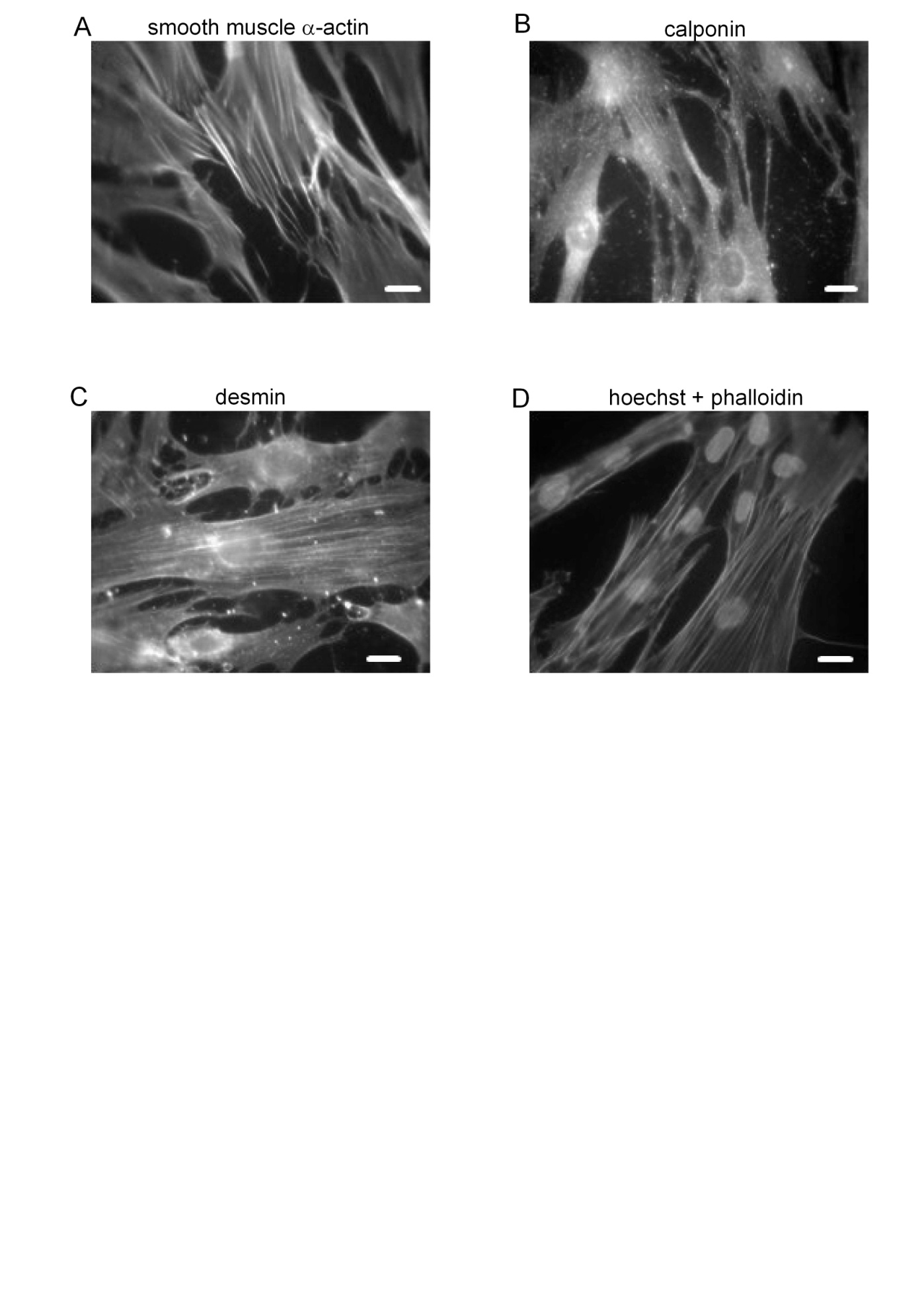
**

Identification of hASMC as smooth muscle by positive staining with anti-smooth muscle α-actin (panel A), anti-desmin (panel B) and anti-calponin (panel C), visualised with Alexa Fluor®488 labelled secondary antibody (Lifetechnologies) and fluorescent microscopy. Cells were also stained with TRITC-labelled phalloidin to confirm the presence of stress fibres in resting cells (Panel D). In Panel D, nuclei are visualised by staining with Hoechst. Scale bar = 20µm.

**Supplementary Figure 2.**

**Effect of PP3 on contractile responses in rat bronchioles.**


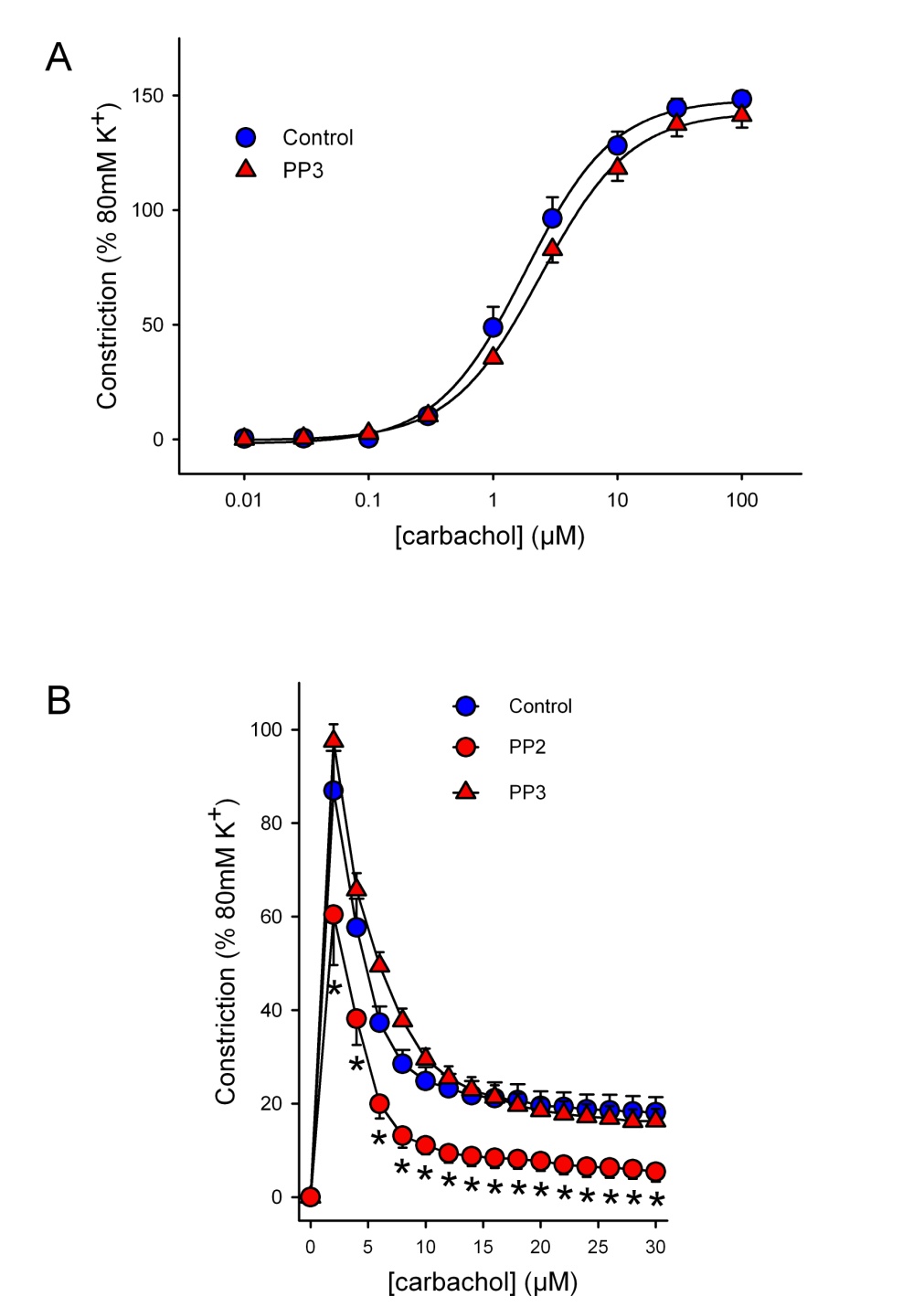


Effects of PP3, the structurally-related negative control for PP2, on the cumulative contractile response to carbachol (A) and the SOCE-associated contraction (B) in rat bronchioles. **A**: Carbachol was applied cumulatively (0.01 – 100µM) at 5min intervals. Two responses were performed in each bronchiole, the second after application of PP3 (30µM, 10min, n=7). Measurements were taken at the end of each 5 min exposure and data fitted by non-linear regression. See results section for EC_50_ values. Data expressed as a % of that induced by 80mM KPSS (mean ± SEM). **B**: SOCE-associated contraction induced by 10µM CPA/200µM EGTA/zero Ca^2+^, followed by reapplication of 2mM Ca^2+^. Mean measurements of amplitude of contractile responses at 2 min intervals after reapplication of Ca^2+^ (± SEM), showing effects of prior incubation with PP3 (30µM, n =8) compared with control response (n =14, taken from Figure 4) and PP2 (30µM, n =9, taken from Figure 4 for comparison). *P<0.05 for PP2 vs. either control or PP3. PP3 caused no significant inhibition compared with control.

**Supplementary Figure 3.**

**Effect of PF-431396 on contractile responses in rat bronchioles.**


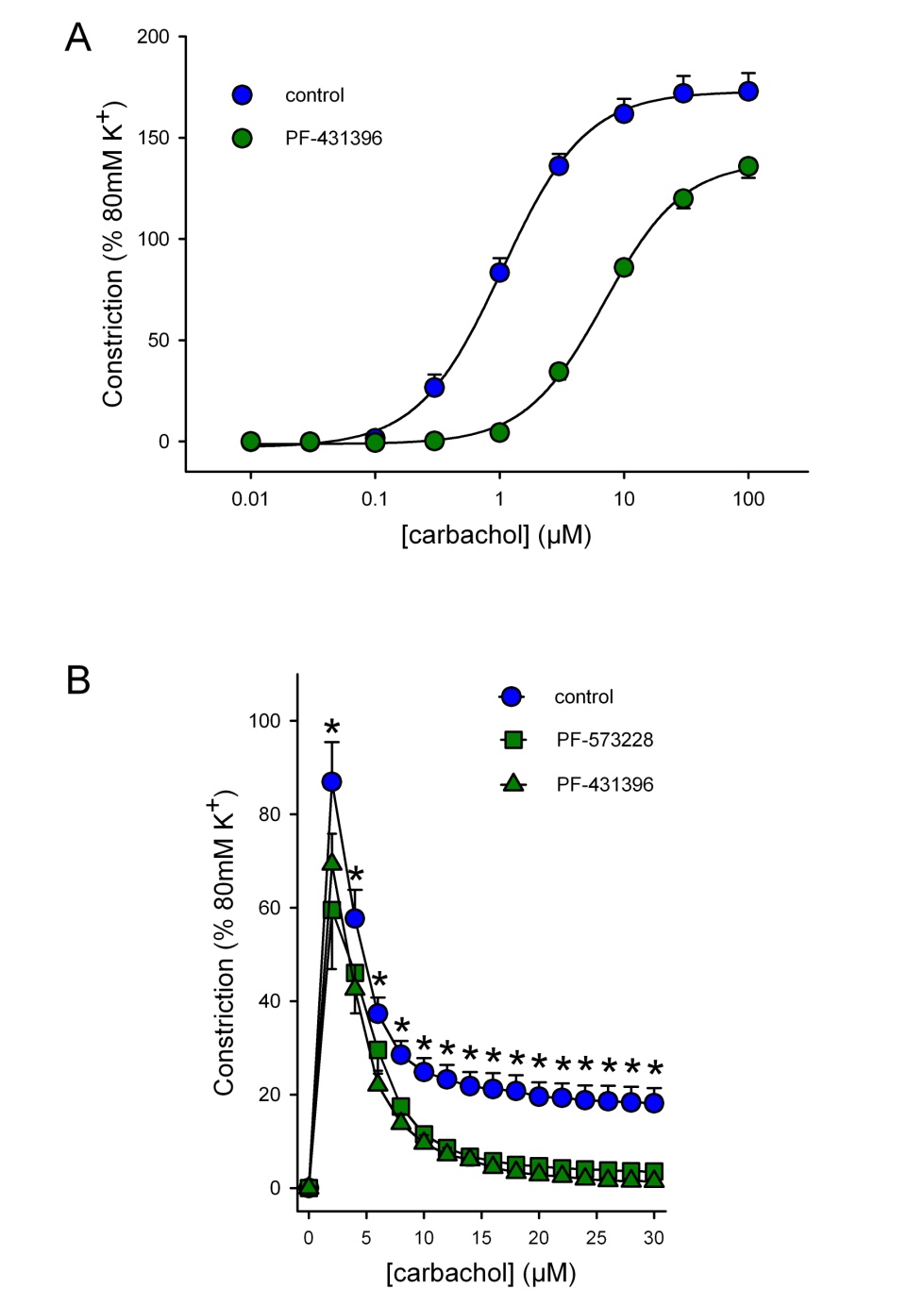


Effects the FAK/PYK2 inhibitor, PF-431396, on the cumulative contractile response to carbachol (A) and the SOCE-associated contraction (B) in rat bronchioles. **A**: Carbachol was applied cumulatively (0.01 – 100µM) at 5min intervals. Two responses were performed in each bronchiole, the second after application of PF-431396 (10µM, 10min, n=7). Measurements were taken at the end of each 5 min exposure and data fitted by non-linear regression. See results section for EC_50_ values. Data expressed as a % of that induced by 80mM KPSS (mean ± SEM). **B**: SOCE-associated contraction induced by 10µM CPA/200µM EGTA/zero Ca^2+^, followed by reapplication of 2mM Ca^2+^. Mean measurements of amplitude of contractile responses at 2 min intervals after reapplication of Ca^2+^ (± SEM), showing effects of prior incubation with PF-431396 (10µM, n =8) compared with control response (n =14, taken from Figure 4) and PF-573228 (10µM, n =9, taken from Figure 4 for comparison). *P<0.05 for either PF-431396 or PF-573228 vs. control. The effects of PF-431396 and PF-573228 were similar.

**Supplementary Figure 4.**

**Effects of SrcFK, Rho-kinase and FAK inhibition on bradykinin-induced contractile responses in rat bronchioles.**

|  | | **Inhibitor** | | |
| --- | --- | --- | --- | --- |
|  |  | **PP2 (n=11)** | **Y27632 (n=4)** | **PF-573228 (n=5)** |
| **High affinity component** | **Max-1**  control  +inhibitor | 12.1 ± 4.1%  1.66 ± 0.5* | 7.23 ± 2.3%  No fit^#^ | 5.98 ± 0.88%  No fit^#^ |
|  | **PD2-1**  control  +inhibitor | -7.08 ± 0.11  -6.70 ± 0.21 | -7.07 ± 0.25  No fit^#^ | -7.42 ± 0.1  No fit^#^ |
| **Low affinity component** | **Max-2**  control  +inhibitor | 24.4 ± 6.0%  6.81 ± 2.0%* | 13.01 ± 3.45%  No fit^#^ | 27.6 ± 4.8%  No fit^#^ |
|  | **PD2-2**  control  +inhibitor | -4.67 ± 0.22  -4.85 ± 0.27 | -5.34 ± 0.54  No fit^#^ | -4.5 ± 0.1  No fit^#^ |

BK concentration responses conducted in the presence or absence of SrcFK inhibitor PP2 (30µM), Rho-kinase inhibitor Y27632 (10µM) or FAK inhibitor PF-573228 (10µM). Maximum induced contractile amplitude (Max) and LogM [EC_50_] (PD2) values derived by non-linear regression fitting to two-site saturation curve. Comparisons by paired t-test: *P<0.05 vs. control. **^#^**No curve fitting possible because of complete or near complete abolition of contraction.
